# Nepotistic Patterns of Violent Psychopathy: Evidence for Adaptation?

**DOI:** 10.3389/fpsyg.2012.00305

**Published:** 2012-08-28

**Authors:** Daniel Brian Krupp, Lindsay A. Sewall, Martin L. Lalumière, Craig Sheriff, Grant T. Harris

**Affiliations:** ^1^Department of Mathematics and Statistics, Queen’s UniversityKingston, ON, Canada; ^2^Department of Psychology, Neuroscience and Behaviour, McMaster UniversityHamilton, ON, Canada; ^3^Department of Psychology, University of LethbridgeLethbridge, AB, Canada; ^4^Department of Psychology, University of SaskatchewanSaskatoon, SK, Canada; ^5^Tulloch Mapping SolutionsOttawa, ON, Canada; ^6^Mental Health CentrePenetanguishene, ON, Canada

**Keywords:** psychopathy, nepotism, kin discrimination, dispersal, mental disorder

## Abstract

Psychopaths routinely disregard social norms by engaging in selfish, antisocial, often violent behavior. Commonly characterized as mentally disordered, recent evidence suggests that psychopaths are executing a well-functioning, if unscrupulous strategy that historically increased reproductive success at the expense of others. Natural selection ought to have favored strategies that spared close kin from harm, however, because actions affecting the fitness of genetic relatives contribute to an individual’s inclusive fitness. Conversely, there is evidence that mental disorders can disrupt psychological mechanisms designed to protect relatives. Thus, mental disorder and adaptation accounts of psychopathy generate opposing hypotheses: psychopathy should be associated with an increase in the victimization of kin in the former account but not in the latter. Contrary to the mental disorder hypothesis, we show here in a sample of 289 violent offenders that variation in psychopathy predicts a *decrease* in the genetic relatedness of victims to offenders; that is, psychopathy predicts an increased likelihood of harming *non-relatives*. Because nepotistic inhibition in violence may be caused by dispersal or kin discrimination, we examined the effects of psychopathy on (1) the dispersal of offenders and their kin and (2) sexual assault frequency (as a window on kin discrimination). Although psychopathy was negatively associated with coresidence with kin and positively associated with the commission of sexual assault, it remained negatively associated with the genetic relatedness of victims to offenders after removing cases of offenders who had coresided with kin and cases of sexual assault from the analyses. These results stand in contrast to models positing psychopathy as a pathology, and provide support for the hypothesis that psychopathy reflects an evolutionary strategy largely favoring the exploitation of non-relatives.

## Introduction

Psychopaths represent a small fraction of the population, but comprise over 15% of incarcerated prisoners and commit approximately half of the most serious crimes (Hare, 1993, 2003). The central features of psychopathy – manipulativeness, impulsivity, callousness, and antisocial behavior – exhibit considerable lifespan stability (Lynam et al., 2007; Witt et al., 2010), and psychopathy is almost always considered a psychiatric or mental disorder associated with clear behavioral, cognitive, affective, and neurophysiological differences (e.g., Blair, 2010; Corr, 2010; Newman et al., 2010).

Classifying psychopaths as disordered, however, requires a satisfactory definition of mental disorder. One influential definition involves the concept of *harmful dysfunction*, where (1) harm to self or others is produced by (2) a mechanism no longer serving its evolved function (Wakefield, 1992). By this definition, evidence that psychopathy has been a viable reproductive strategy during human evolution would mitigate against the view that psychopathy is a mental disorder. One leading hypothesis regarding the evolution of psychopathy is that it is an “alternative” reproductive strategy whereby a small number of individuals take advantage of their more populous, cooperative counterparts by extracting material, sexual, and perhaps reputational resources through the use of deception and coercion (Harpending and Sobus, 1987; Mealey, 1995; Harris et al., 2001a).

There is evidence consistent with the argument that psychopaths have pursued a frequency-dependent strategy that increased reproductive success in ancestral environments through persistent social exploitation (Harpending and Sobus, 1987; Mealey, 1995; Harris et al., 2001a; Lalumière et al., 2001, 2008), and attempts to disconfirm this functional hypothesis have not been successful. First, psychopathy is neither comorbid nor associated with the neurodevelopmental perturbations characteristic of other serious mental illnesses, such as psychosis and mental retardation (Harris et al., 2001a, 2007b; Lalumière et al., 2001). Second, though psychopathy is negatively associated with performance in social exchange reasoning (Ermer and Kiehl, 2010) and measures of emotional intelligence (Ermer et al., 2012), psychopaths have an intact Theory of Mind (Richell et al., 2003) and psychopathy is positively associated with performance on a social exchange task (the Prisoner’s Dilemma; Mokros et al., 2008), accuracy in the detection of emotions (Book et al., 2007), and accuracy in the prediction of victim vulnerability (Wheeler et al., 2009). Third, psychopathy is positively associated with mating effort, precocious and coercive sexuality, sexually targeting reproductively viable victims, and sexual arousal to coercion (i.e., psychopaths are more genitally aroused to coercive sex; Quinsey et al., 1995; Lalumière and Quinsey, 1996; Harris et al., 2007b; reviewed in Lalumière et al., 2005). Finally, while faring more poorly in almost every life domain, psychopathic offenders appear to have at least as many offspring as others (Harris et al., 2007b; Pulkkinen et al., 2009; Vachon et al., 2012), and are more likely to offend in instrumental, or goal-directed ways (Williamson et al., 1987; Cornell et al., 1996). Thus, despite many apparently self-defeating traits, evidence suggests that psychopathy has persisted in human lineages because of its historical success in terms of fitness. By this adaptationist account, psychopaths are expected to lead selfish lives, increasing fitness by taking large risks to acquire resources.

A behavioral strategy – such as psychopathy, we suggest – can serve an individual’s genetic interests, but genetic success does not depend merely upon one’s own survival and reproduction; it also depends upon the survival and reproduction of *others* carrying copies of one’s alleles (Hamilton, 1964). Genes underlying behavioral differences are likely to be carried by genealogical relatives. Thus, by reducing the costs of an action to relatives and imposing them instead on non-relatives, individuals can improve their *inclusive fitness* prospects via the reproduction of copies of their alleles housed in the bodies of their kin whom, when lineally descended, influence direct fitness and, when related collaterally, influence indirect fitness. As such, individuals executing well-designed strategies, a necessary feature of psychological adaptations, should tend to be nepotistic – providing aid to close genealogical kin and/or sparing them from harm.

It is abundantly clear that humans are generally nepotistic. For example, genetic relatives are far less subject to violence and aggression than are others (Daly and Wilson, 1988a,b; Harris et al., 2007a). Indeed, violence toward genealogical kin is positively associated with psychiatric illness (Daly and Wilson, 1988a), and harming one’s genetic relatives is associated with an increased likelihood that an offender will be regarded as mentally disordered and excused from punishment (Harris et al., 2007a). Thus, psychopathy-as-mental-disorder and psychopathy-as-adaptation conceptualizations generate opposing hypotheses with respect to the association between psychopathy and nepotistic inhibition of violence: variation in psychopathy should be associated with an increased risk of violence toward genealogical kin in the former case but not in the latter. That is, psychopaths should be less discriminating (with respect to genetic relatedness) in the targets of their violence than mentally healthy individuals on the mental disorder hypothesis, but they should be at least as discriminating as healthy individuals on the adaptation hypothesis.

Here, we investigated whether psychopathy is associated with patterns of nepotistic inhibition in violence among a sample of 289 violent offenders. The mental disorder hypothesis of psychopathy predicts a positive association between variation in psychopathy and victim-offender relatedness, whereas adaptation hypotheses predict no association, or even possibly a negative association (see below), between psychopathy and victim-offender relatedness. To anticipate, we find that variation in psychopathy is *negatively* associated with the risk of harm to genealogical kin, and so we examined two of the general processes by which psychopaths may achieve nepotistic behavioral patterns. By design, *de facto* nepotism may be caused by dispersal, whereby exploitative individuals migrate away from their natal “patch” and thereby reduce harm to relatives still residing there (Hamilton and May, 1977; Frank, 1986; El Mouden and Gardner, 2008). Using data from birth, family residence, and offense locations, we tested whether variation in psychopathy is associated with patterns of dispersal that could account for the association between psychopathy and nepotistic inhibition.

Alternatively, nepotism may be caused by kin discrimination, whereby organisms treat conspecifics differently as a function of genetic relatedness via some form of kin recognition (reviewed in Krupp et al., 2011). One function of kin recognition is inbreeding avoidance (reviewed in Pusey and Wolf, 1996) and, given that psychopathic offenders are more likely to commit sexual offenses than are non-psychopathic offenders (Harris et al., 2003, 2007b), it is possible that psychopathy is negatively associated with victim-offender relatedness because of psychological mechanisms tailored by selection to minimize the effects of inbreeding depression – the elevated fitness costs of mating with close genetic relatives (Charlesworth and Charlesworth, 1999; Penn and Potts, 1999). To test this, we examined whether the negative association between psychopathy and nepotistic inhibition holds after removing all incidents of sexual assault.

## Materials and Methods

Data were gathered from a review of 400 case files of violent male offenders evaluated at the Mental Health Centre, Penetanguishene, Canada. These cases have been reported in previous studies that established that methods of measuring psychopathy and scoring research variables were valid and highly reliable (e.g., Harris et al., 1993, 2003). These studies yielded inter-rater reliabilities for the psychopathy measure (see below) higher than typically reported (e.g., Harris et al., 2003 reported a Pearson *r* = 0.95), the same factor structure (Harris et al., 1994, 2007b), and higher predictive validities for violence than typically reported (e.g., Harris et al., 1993 reported a Pearson *r* = 0.34). These studies reported that these offenders, on average, had prior histories of childhood aggression, juvenile and adult criminality, incarceration, and substance abuse. A minority met the diagnostic criteria for affective disorder, mental retardation, or schizophrenia, and most were briefly evaluated before serving correctional sentences.

From the larger samples, we excluded those for whom psychopathy could not be assessed due to incomplete information (<15%). Cases were also excluded when the file had been discarded, birth was outside of Canada, locations of birth or index offense (the offense resulting in admission) were unstated or less specific than municipality, victim-offender relationships were unspecified, or the index offense was committed in more than one location. Cases in which an offender had more than one victim of the same type (e.g., non-relative; see below) were kept in the dataset; each case nonetheless contributed a single data point to any given analysis^1^. Psychopathy was measured by the Psychopathy Checklist-Revised (PCL-R; Hare, 1991), a 20-item scale completed from case files by raters trained through workshops and supervised practice. Scoring from case files alone, while reliable and valid (see above), might underestimate the number of individuals who are “truly” psychopathic (Wong, 1988) and so, based on suggestions by Wong (1988) and Harris et al. (1994), we classified as psychopaths those offenders scoring 25 or greater on the PCL-R. The final sample consisted of 289 offenders, 109 of whom were classified as psychopaths. Victim-offender relationship was coded for the index offense only. Scoring of the PCL-R was blind to study hypotheses and dispersal distances but not to index offense details.

Victim-offender relatedness was coded as first-degree relative (full sibling, biological parent, or biological child; *n* = 32), second-degree relative (aunt, uncle, half-sibling, or grandparent; *n* = 9), or non-relative (e.g., spouse, friend, acquaintance, stepchild; *n* = 248). The numerical values assigned to these three categories of relatedness were 1, 2, and 3, respectively. The victims in three additional cases were noted as “close family” in the files but did not specify any genetic relationship. Because such cases may refer to non-genealogical kin (e.g., spouse or stepchild), they were not included in the analysis.

The geographic locations of each offender’s place of birth and place of index offense were coded as coordinate degrees longitude and latitude using Wikipedia entries. Dispersal data were determined by computing the great circle distance between these two points. (Driving distances were also calculated using Google Maps. However, the correlation between coordinate-based, “as the crow flies,” distances and driving distances is *r* = 0.995, *p *< 0.001, so analyses using this measure would have been redundant with those reported below.)

To test the predicted associations between psychopathy and harm delivered by offenders to their genealogical kin, we performed ordered probit regressions (Aitchison and Silvey, 1957; McKelvey and Zavoina, 1975) of victim-offender relatedness on PCL-R score. In addition to the chi-square tests and associated *p*-values for each regression model, Nagelkerke *R*^2^ values are reported; this latter statistic is a measure of the improvement of the fitted model over the null model (Nagelkerke, 1991). However, as both the mental disorder and adaptation hypotheses of psychopathy considered above make only directional predictions about the association between PCL-R score and victim-offender relatedness, and not predictions about the improvement in model fit, Nagelkerke *R*^2^ values are presented simply for the sake of completeness.

To test the hypothesized association between psychopathy and dispersal, we computed Spearman’s rho (*r*_s_) coefficients between PCL-R score and distance dispersed (of the offenders and of the offenders’ kin). Additionally, we performed chi-square tests to investigate differences between psychopathic (PCL-R ≥ 25) and non-psychopathic offenders (PCL-R < 25) in the frequencies of dispersal and coresidence with kin. All statistical tests were two-tailed (α = 0.05).

## Results

**Figure 1:**
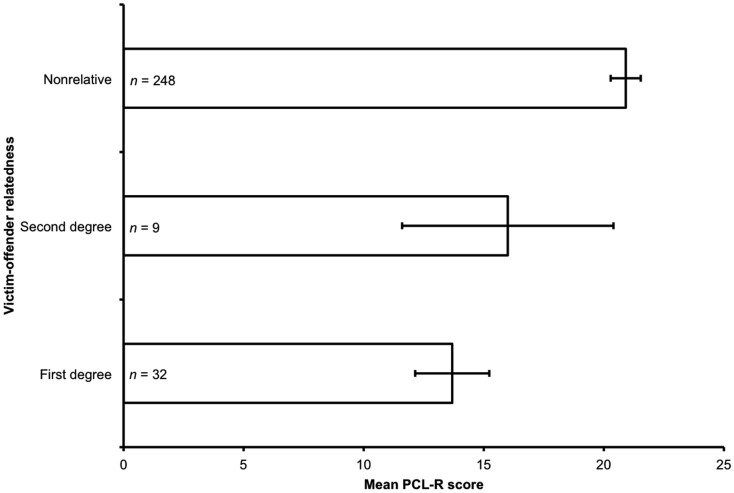
**Mean PCL-R score and victim-offender relatedness**. The figure shows the mean PCL-R score for all offenders meeting inclusion criteria for each category of victim-offender relatedness: first-degree relatives; second-degree relatives; and non-relatives. Error bars represent standard errors of the mean. There was a significant effect of PCL-R score on victim-offender relatedness (Nagelkerke *R*^2^ = 0.06, χ^2^ = 17.97, *p *< 0.001): as PCL-R scores increased, victim-offender relatedness decreased.

PCL-R score significantly predicted victim-offender relatedness (Figure 1): as PCL-R score increased, victim-offender relatedness decreased (ordered probit regression: Nagelkerke *R*^2^ = 0.06, χ^2^ = 17.97, *p *< 0.001).

**Figure 2:**
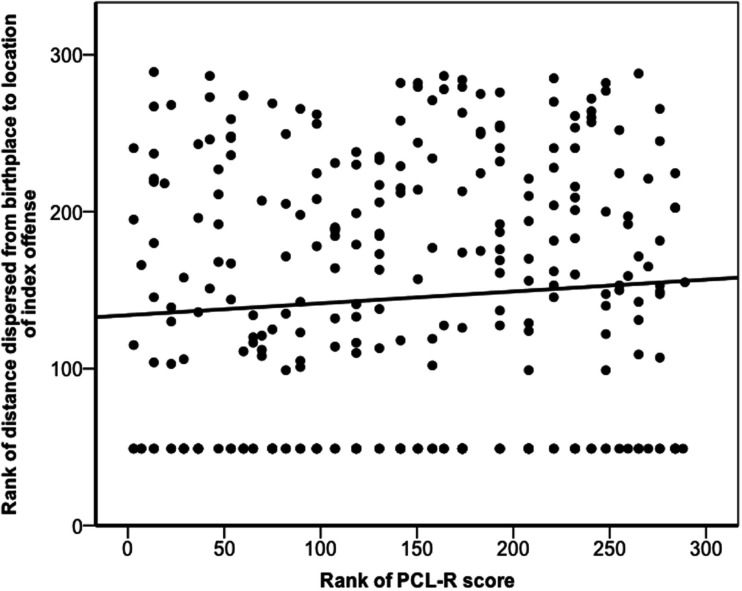
**Dispersal of offender as a function of offender’s PCL-R score**. The figure depicts the ranked distances that offenders dispersed from their places of birth to the locations of their index offenses against their ranked PCL-R scores. The solid line represents a linear regression line of best fit for the ranked data (*r*_s_ = 0.08, *p *= 0.193).

Dispersal was not normally distributed (Shapiro–Wilk *W* = 0.556, *p *< 0.001). PCL-R score and the distance an offender dispersed were not significantly correlated (*r*_s_ = 0.08, *p *= 0.193; Figure 2). However, one might construe the dispersal question differently: given an offender “dispersed,” what was the probability that he was a psychopath? We defined dispersing offenders as those who committed index offenses more than 5 km from their birthplaces: 41% of dispersing individuals and 32% of non-dispersing individuals qualified as psychopathic, but these values did not significantly differ (χ^2^ = 2.06, *p* = 0.151).

Although psychopaths did not disperse significantly further, or more often, from their birthplaces than did non-psychopaths, they may have dispersed more locally, away from their genealogical kin but still in close proximity; alternatively, their kin may have themselves dispersed. We analyzed the dispersal of offenders’ genealogical relatives (relatives other than those victimized by the offenders), coded as related versus unrelated, from the locations of offenders’ index offenses to the offenders’ relatives’ residences, where locations were available and conformed to the inclusion criteria above (i.e., no cases with kin residing outside of Canada, no cases with kin for whom location data were less specific than municipality). The relatives of 129 offenders were retained; where more than one residence was listed, we used the arithmetic mean of these locations as the residence location. Dispersal among the relatives of offenders was not normally distributed (Shapiro–Wilk *W* = 0.426, *p *< 0.001). PCL-R score and the distance an offender’s kin dispersed were not significantly correlated (Spearman’s rho: *r*_s_ = 0.14, *p *= 0.121).

Psychopaths, however, were less likely than non-psychopaths to have coresided with kin prior to committing their offenses (χ^2^ = 7.84, *p* = 0.005). To test whether coresidence could explain the apparent effect of psychopathy on victim-offender relatedness, we removed the 49 cases in which offenders coresided with genealogical kin and examined again whether there remained evidence of nepotistic inhibition in the violent offenses of psychopaths. In this subsample (*n* = 240), PCL-R score marginally predicted victim-offender relatedness: victim-offender relatedness decreased as PCL-R score increased (ordered probit regression: Nagelkerke *R*^2^ = 0.02, χ^2^ = 5.48, *p* = 0.065). There was again no significant association between PCL-R score and the distance an offender dispersed (Spearman’s rho: *r*_s_ = 0.00, *p* = 0.993) or between PCL-R score and the distance an offender’s kin dispersed (relatives other than those victimized by the offenders; Spearman’s rho: *r*_s_ = 0.07, *p* = 0.487).

Taking again the complete sample (*n* = 289), there were 53 cases in which the primary victim had been sexually assaulted, and five of these involved genetic relatives. Psychopathic offenders were significantly more likely than non-psychopathic offenders to have committed sexual assault (χ^2^ = 16.65, *p *< 0.001). After removing all cases of sexual assault (*n* = 236), PCL-R score remained significantly associated with victim-offender relatedness (ordered probit regression: Nagelkerke *R*^2^ = 0.08, χ^2^ = 18.90, *p *< 0.001): victim-offender relatedness again decreased as PCL-R score increased.

## Discussion

In light of Wakefield’s (1992) definition of mental disorder, evidence that psychopathy retains nepotistic design features is at odds with psychopathy being a mental disorder, given that a diagnosis of mental disorder tends to be positively associated with the victimization of genealogical kin (Daly and Wilson, 1988a; Harris et al., 2007a). The present findings suggest that psychopathy leads to an *increase* in nepotistic inhibition among violent offenders – significantly so in the overall sample, and marginally so in the subsample of offenders who did not coreside with kin. Thus, our central result fails to support the hypothesis that psychopathy is a pervasively disruptive mental disorder. This result is especially surprising given that psychopaths tend to be ruthlessly violent, impulsive, and lacking in empathy. Nepotistic inhibition of violence is, however, predicted by hypotheses that psychopathy was an adaptive strategy designed by selection to increase short-term, direct fitness interests, so long as strategic gains did not come at greater expense to indirect fitness. Although their behavior is objectionable, psychopaths appear to pursue a nepotistic strategy that could have helped to advance their reproductive interests in ancestral environments, much like “normally” functioning humans.

Psychopaths are said to lead transient lives (e.g., Hare, 1993, p. 168). In our study, psychopathy was not significantly associated with the distance an offender or his kin dispersed, though psychopathic offenders were less likely than non-psychopathic offenders to coreside with genealogical kin. This could suggest that psychopaths avoided harming their relatives by leaving the home, or perhaps that kin more readily ejected psychopathic individuals from the home. After removing offenders who coresided with kin, however, psychopathy remained marginally negatively associated with victim-offender relatedness. Thus, our failure to support the mental disorder hypothesis cannot be ascribed to differential access between psychopathic and non-psychopathic offenders. To maintain levels of nepotistic inhibition similar to non-psychopaths, violent psychopaths might make use of kin discrimination mechanisms, such as those based on within-household association (Lieberman et al., 2007) and phenotypic similarity (DeBruine, 2002; Krupp et al., 2008). Direct tests of kin discrimination mechanisms are possible (Krupp et al., 2011), and would be enlightening in this case.

In the interim, consideration of the effects of inbreeding depression over human evolution can provide an indirect test of the involvement of kin discrimination mechanisms in the relationship between psychopathy and nepotistic inhibition reported here. Inbreeding depression has selected for a psychology of inbreeding avoidance that relies on many of the same mechanisms as those proposed herein to explain nepotistic inhibition among psychopathic offenders (i.e., kin recognition and dispersal; see Pusey and Wolf, 1996; Lieberman et al., 2007; DeBruine et al., 2011; Krupp et al., 2011). Moreover, inbreeding depression affects the fitness of genetic relatives, and so inbreeding avoidance can be understood in part as a form of nepotistic inhibition. As psychopathy is associated with an increased likelihood of committing sexual assault (Harris et al., 2003, 2007b), it is possible that psychopathy is negatively associated with victim-offender relatedness because the crimes of psychopaths more frequently activate inbreeding avoidance responses. Analysis of the data, however, does not support this hypothesis: whereas psychopathic offenders were disproportionately more likely than their non-psychopathic counterparts to have committed sexual assault, PCL-R score continued to be significantly associated with a decline in victim-offender relatedness after all cases of sexual assault had been removed from the analyses.

### Limitations

The current study made use of PCL-R data that were scored without a structured interview. Although a structured interview is a standard part of the scoring procedure, the PCL-R remains a reliable measure of psychopathy when scored without it (Wong, 1988; Hare, 2003), and we have presented evidence elsewhere that our methods were at least as reliable and valid as those including interviews (e.g., Harris et al., 1993, 2003). However, PCL-R scores derived without the interview might underestimate the number of individuals that would actually score highly with the interview (Wong, 1988). To remedy this concern, we used a lower criterion score (PCL-R ≥ 25) in the dichotomous (chi-square) analyses than is conventionally used. We derived this value from Wong (1988) and from previous research suggesting that the PCL-R ≥ 25 criterion captures an effectively “pure” group of psychopaths (Harris et al., 1994); this value has been used in numerous other studies (e.g., Harris et al., 1991, 2001a; Rice et al., 1992). The pattern of results is unchanged when we replace the PCL-R ≥ 25 criterion with a PCL-R ≥ 30 criterion, however, and the criterion has no impact on the continuous (regression and correlational) analyses, in any case.

The present study relied on index offenses. These represented only a sample of all of the adjudicated and undetected offenses the offenders ever committed. Relying on such a sample of offenses is inherent in almost all research on offender populations and, certainly, the present sample (and the adjudicated offending record overall) is unlikely to be a random sample of all lifetime criminal and antisocial behavior; for example, the present sample is likely to represent generally more serious crime. While we are not aware of any findings about psychopathy, differences between index and historical offenses, or differences between detected and undetected crime that lead us to think that our findings would have been different had we known about offenders’ total lifetime criminality rather than just their index offenses, this potential limitation must be acknowledged and evaluated in future studies. It is possible, for instance, that the cumulative effects of psychopathy on an offender’s kin, including negative reputational effects, are substantial, even though the costs of individual actions may be small. Thus, an attempted replication of our findings over a longer offense history could prove to be important.

Likewise, the data available to us do not provide information about the causes of dispersal. Plausibly, psychopathic individuals drive their kin from their homes (rather than the converse), which would explain why psychopathic offenders in our sample were significantly less likely to coreside with kin. It strikes us, however, that individuals driven from their homes would also have a greater propensity to be driven further afield. Were this the case, we would expect a positive association between PCL-R score and the dispersal of an offender’s kin, but this association was not statistically significant. Thus, while we cannot strictly rule this phenomenon out, we do not think it particularly likely.

### Alternative accounts to “nepotism”

Our account here of psychopathy as a selfish or perhaps spiteful (see below) strategy is a functional argument about the historical reproductive consequences of individuals bearing psychopathic traits. Hence, it cannot be pitted against a purely mechanistic account of precisely *how* psychopathic individuals’ relatives are spared, as functional and mechanistic arguments pertain to different levels of analysis, and are thus complementary (Tinbergen, 1963; Scott-Phillips et al., 2011). Indeed, our functional account led to the generation of two broad mechanistic hypotheses, namely that psychopaths are *de facto* nepotists by virtue of (1) differential patterns of dispersal and (2) their use of kin recognition systems.

Nevertheless, it is reasonable to ask whether the pattern of results suggesting nepotism can be explained without any appeal to nepotistic design *per se*. For instance, features of psychopathy include criminal versatility – because they commit more crimes, psychopaths are more likely to engage in a wide variety of criminal behaviors – and a lack of empathy and, presumably, feelings of love. While such features of psychopathy may be the proximate manifestations of a functional strategy designed to exploit primarily non-relatives, they may alternatively cause, *as by-products*, the patterns of nepotistic inhibition discovered here. Could it be that psychopaths are less likely to harm genealogical relatives, not because of any evolved mechanisms designed to protect relatives from harm, but for other reasons entirely?

First, psychopathic offenders are more criminally versatile than non-psychopathic offenders (Hare, 1991, 2003). Thus, it is possible that psychopathic offenders are apparently nepotistic only because they are busy committing many other sorts of crimes. However, our analysis was restricted to violent offenses; hence, “versatility” could only pertain in this case to variation in victim choice and location. As discussed above, however, genealogical relatives are grossly under-represented as victims of violence (Daly and Wilson, 1988a,b; Harris et al., 2007a). Thus, a “versatile” offender (in the sense of victim choice) is expected to be *more* likely to harm kin than a less versatile offender. Yet, we found the opposite: psychopathy negatively predicted victim-offender relatedness. Likewise, we did not find any significant association between psychopathy and distance or frequency of dispersal, and PCL-R score continued to remain marginally negatively associated with victim-offender relatedness after removing offenders who had coresided with their kin. Thus, “versatility” in the domain of victim location also does not seem to be a plausible explanation for our results.

Second, psychopaths do not feel empathy (Hare, 1991, 2003) and are presumably unable to feel love for others. Perhaps, then, they are unlikely to harm relatives simply because their emotional systems are aberrant. Research on violence, however, suggests that emotions such as love are *protective* in the case of genetic relatives; that is, normal people tend to spare their genetically related loved ones from harm (Daly and Wilson, 1988a,b; Harris et al., 2007b). Thus, as above, one would again predict that individuals lacking loving or empathic sentiments would be more likely to harm their kin, but we found the opposite in the case of psychopathic offenders.

A third alternative account regards the possibility that psychopaths better avoid prosecution for crimes against relatives because they are better at intimidating, deceiving, or otherwise manipulating their kin than are non-psychopaths, and so their kin are less likely to report an offense. On this hypothesis, one would expect that only psychopaths coresiding with their kin, and who thus may have better opportunities to exercise manipulation, would be less likely to have convictions against relatives, but again this was not the case. In our study, there remained a marginally negative association between PCL-R score and victim-offender relatedness among offenders who did not coreside with kin. Thus, the general pattern of results with regard to psychopathic offenders remained the same whether individuals with the greatest access to their relatives, for the purposes of manipulation, were included or were not.

Fourth, substance abuse may have the effect of dysregulating adaptive systems, such as mechanisms of kin recognition and social motivation, that protect genetic relatives from harm. Thus, a difference in substance abuse rates between groups could explain the apparent nepotism seen in the study. However, substance abuse rates have previously been found to be significantly higher among psychopathic offenders than among non-psychopathic offenders (Rice and Harris, 1995). Hence, any effect of substance abuse on the dysregulation of nepotistic systems should reduce the appearance of nepotism among psychopathic offenders rather than inflate it.

Finally, psychopathy tends not to be comorbid with the neurodevelopmental perturbations associated with various forms of psychosis (schizophrenia or major affective disorders; Harris et al., 2001a), so there may have been an excess number of psychotic individuals within the non-psychopathic group. Similarly to the preceding argument, then, there may have been an *a priori* bias working against the mental disorder hypothesis of psychopathy. That is, psychopaths may appear to be avoiding harming relatives simply because the non-psychopathic group has a preponderance of individuals that are nepotistically disinhibited as a consequence of psychosis-induced mental dysregulation. However, serious consideration shows that the mental disorder hypothesis does not lend itself to this argument: if psychopaths are mentally disordered, then our study has instead “stacked the deck” in favor of this hypothesis, because *all* individuals in the psychopathic group would be disordered and thus suffer from any potential dysregulation of nepotistic design, whereas only *some* of the individuals in the non-psychopathic group would likewise suffer the same dysregulatory issues. Thus, any partial bias caused by psychosis among non-psychopaths might plausibly reduce any predicted negative effect of psychopathy on nepotistic inhibition, but it would not be expected to eliminate it, let alone reverse it, as our results indicate.

### Future directions

The concept of an adaptation is an onerous one (Williams, 1966). To be clear, we do not claim to have demonstrated unequivocally that psychopathy is an adaptation. Rather, we believe that our findings fail to support the conceptualization of psychopathy as a mental disorder and instead provide support for the notion that psychopathy may, in some form or another, have an adaptive function. Our findings fit well within the body of work showing that psychopathy: (1) is not associated with the neurodevelopmental perturbations characteristic of other serious mental disorders (Harris et al., 2001a, 2007b; Lalumière et al., 2001); (2) is positively associated with successful social exploitation (Mokros et al., 2008), the detection of emotions (Book et al., 2007), and the prediction of victim vulnerability (Wheeler et al., 2009); (3) is positively associated with mating effort and related patterns of sexual behavior (Quinsey et al., 1995; Lalumière and Quinsey, 1996; Harris et al., 2007b); (4) shows no negative (and shows perhaps even a positive) effect on reproductive success (Harris et al., 2007b; Pulkkinen et al., 2009; Vachon et al., 2012); and (5) is associated with an increased likelihood of offending in instrumental ways (Williamson et al., 1987; Cornell et al., 1996). Nonetheless, further research testing hypotheses of “special design” will be necessary to continue to build the case for psychopathy as an adaptation.

Whether properly construed as adaptation or pathology, the genetic architecture underlying psychopathy is almost certainly multi-locus and multi-allelic (reviewed in Harris et al., 2001b; see also Lalumière et al., 2008). Evidence of significant heritability is to be expected by most models of psychopathy, which tend to posit the phenomenon as being under frequency-dependent selection (e.g., Harpending and Sobus, 1987; Mealey, 1995). If numerous genes of small effect cause psychopathy, some individuals may inherit a larger “dose” of the alleles promoting the development of a psychopathic personality than others. As such, there may exist one or more optimal doses of psychopathic alleles; individuals inheriting too large or small a dose would pay fitness costs relative to those with the optimal doses (see Nettle, 2004, for a similar argument regarding depression). Thus, it is possible that many individuals bearing psychopathic traits are not disordered, but that those with extremely psychopathic traits are. Further research on the psychological and behavioral consequences of psychopathy, in tandem with molecular genetics methods, may provide important clues to the questions of adaptation and optimal dosage of genes favoring psychopathic tendencies.

Interestingly, our findings are consistent with an alternative functional account of psychopathy that captures puzzling aspects not otherwise well explained by the account that psychopathy is an evolutionarily selfish strategy – that is, a strategy designed to increase the direct fitness of individuals playing it by decreasing the fitness of others. Psychopaths are especially willing to perform acts of physical violence (Harris et al., 1991; Serin, 1991), particularly against male strangers (Williamson et al., 1987). It is unclear, however, what psychopaths might have gained from such violence, as the response by others would likely have been dire in ancestral (as well as contemporary) environments. More puzzling, psychopaths are relatively undeterred by the threat or application of punishment, and recidivate at considerably higher rates than do non-psychopathic offenders (Harris et al., 1991).

A purely selfish strategy would neither be expected to impose pointless harm on male non-relatives nor to incur the large costs of such actions. The infliction and acceptance of such costs, however, are hallmarks of *spiteful* strategies (Hamilton, 1970). Spiteful behavior can evolve when harm is directed at individuals who are significantly *less* likely than chance to bear copies of the alleles associated with the behavior – so-called “negative” relatives – because it decreases the reproductive success of rival alleles housed in the bodies of others (Gardner and West, 2004). Selection can favor strategies that entail costs to direct fitness if their indirect fitness benefits outweigh these costs (Hamilton, 1964, 1970). In this case, the indirect fitness benefit comes from reducing the fitness of rival alleles, thereby increasing the relative success of copies of the spiteful individual’s own alleles. Perhaps psychopaths execute a more complex strategy than simple selfishness: they behave selfishly where they can improve their direct reproduction, engaging, for instance, in sexual coercion, but in instances where causing injury entails direct costs, they may behave spitefully, systematically imposing costs on negative relatives. As discussed above, selection may produce *de facto* nepotism by designing individuals to disperse from their natal “patch” when engaging in competitive behavior (Hamilton and May, 1977; Frank, 1986; El Mouden and Gardner, 2008) and to discriminate among social partners based on cues of relatedness (Krupp et al., 2011, 2012). Research on psychopathy should explore this possibility further by investigating (1) the genetic relatedness of the victims of psychopathic offenders, (2) the causes and consequences of dispersal, and (3) the abilities of psychopaths to discriminate cues of positive and negative relatedness and their attendant consequences (Krupp et al., 2011, 2012).

## Conflict of Interest Statement

The authors declare that the research was conducted in the absence of any commercial or financial relationships that could be construed as a potential conflict of interest.

## Footnotes

^1^Of the 289 cases that met the inclusion criteria, five had victims of different relatedness categories (e.g., victim 1 was a first-degree relative and victim 2 was a non-relative). In these cases, we coded the relatedness of the victim to the offender as that of the first (primary) victim listed in the incident file. Reanalyzing the data after excluding these five cases does not affect the general pattern of results or their statistical significance.

## References

[B1] Aitchison J. Silvey S. D. (1957). The generalization of probit analysis to the case of multiple responses. Biometrika 44, 131–140 10.1093/biomet/44.1-2.131

[B2] Blair R. J. R. (2010). Psychopathy, frustration, and reactive aggression: the role of ventromedial prefrontal cortex. Br. J. Psychol. 101, 383–399 10.1348/000712609X418480 19321035

[B3] Book A. S. Quinsey V. L. Langford D. (2007). Psychopathy and the perception of affect and vulnerability. Crim. Justice Behav. 34, 531–542 10.1177/0093854806293554

[B4] Charlesworth B. Charlesworth D. (1999). The genetic basis of inbreeding depression. Genet. Res. 74, 329–340 10.1017/S0016672399004152 10689809

[B5] Cornell D. G. Warren J. Hawk G. Stafford E. Oram G. Pine D. (1996). Psychopathy in instrumental and reactive violent offenders. J. Consult. Clin. Psychol. 64, 783–790 10.1037/0022-006X.64.4.783 8803369

[B6] Corr P. J. (2010). The psychoticism–psychopathy continuum: a neuro psychological model of core deficits. Pers. Indiv. Differ. 48, 695–703 10.1016/j.paid.2009.12.023

[B7] Daly M. Wilson M. (1988a). Homicide. Hawthorne, NY: Aldine de Gruyter

[B8] Daly M. Wilson M. (1988b). Evolutionary social psychology and family homicide. Science 242, 519–524 10.1126/science.3175672 3175672

[B9] DeBruine L. M. (2002). Facial resemblance enhances trust. Proc. Biol. Sci. 269, 1307–1312 10.1098/rspb.2002.2034 12079651 PMC1691034

[B10] DeBruine L. M. Jones B. C. Watkins C. D. Roberts S. C. Little A. C. Smith F. G. Quist M. C. (2011). Opposite-sex siblings decrease attraction, but not prosocial attributions, to self-resembling opposite-sex faces. Proc. Natl. Acad. Sci. U.S.A. 108, 11710–11714 10.1073/pnas.1105919108 21709272 PMC3136321

[B11] El Mouden C. Gardner A. (2008). Nice natives and mean migrants: the evolution of dispersal-dependent social behavior in viscous populations. J. Evol. Biol. 21, 1480–1491 10.1111/j.1420-9101.2008.01614.x 18811663

[B12] Ermer E. Kahn R. E. Salovey P. Kiehl K. A. (2012). Emotional intelligence in incarcerated men with psychopathic traits. J. Pers. Soc. Psychol. 103, 194–204 10.1037/a0027328 22329657 PMC3378803

[B13] Ermer E. Kiehl K. A. (2010). Psychopaths are impaired in social exchange and precautionary reasoning. Psychol. Sci. 21, 1399–1405 10.1177/0956797610384148 20855897 PMC3042879

[B14] Frank S. A. (1986). Dispersal polymorphisms in subdivided populations. J. Theor. Biol. 122, 303–309 10.1016/S0022-5193(86)80122-9 3626575

[B15] Gardner A. West S. A. (2004). Spite and the scale of competition. J. Evol. Biol. 17, 1195–1203 10.1111/j.1420-9101.2004.00775.x 15525404

[B16] Hamilton W. D. (1964). The genetical evolution of social behaviour (I and II). J. Theor. Biol. 7, 1–52 10.1016/0022-5193(64)90039-6 5875341

[B17] Hamilton W. D. (1970). Selfish and spiteful behaviour in an evolutionary model. Nature 228, 1218–1220 10.1038/2281218a0 4395095

[B18] Hamilton W. D. May R. M. (1977). Dispersal in stable habitats. Nature 269, 578–581 10.1038/269578a0

[B19] Hare R. D. (1991). Manual for the Hare Psychopathy Checklist-Revised. Toronto: Multi-Health Systems

[B20] Hare R. D. (1993). Without Conscience. New York: Guilford Press

[B21] Hare R. D. (2003). Manual for the Hare Psychopathy Checklist-Revised (2nd edition). Toronto: Multi-Health Systems

[B22] Harpending H. C. Sobus J. (1987). Sociopathy as an adaptation. Ethol. Sociobiol. 8(Suppl. 1), 63–72 10.1016/0162-3095(87)90019-7

[B23] Harris G. T. Hilton N. Z. Rice M. E. Eke A. W. (2007a). Children killed by genetic parents versus stepparents. Evol. Hum. Behav. 28, 85–95 10.1016/j.evolhumbehav.2006.08.001

[B24] Harris G. T. Rice M. E. Hilton N. Z. Lalumière M. L. Quinsey V. L. (2007b). Coercive and precocious sexuality as a fundamental aspect of psychopathy. J. Pers. Disord. 21, 1–27 10.1521/pedi.2007.21.1.1 17373887

[B25] Harris G. T. Rice M. E. Cormier C. A. (1991). Psychopathy and violent recidivism. Law Hum. Behav. 15, 625–637 10.1007/BF01065856 12182529

[B26] Harris G. T. Rice M. E. Lalumière M. (2001a). Criminal violence: the roles of psychopathy, neurodevelopmental insults, and antisocial parenting. Crim. Justice Behav. 28, 402–426 10.1177/009385480102800402

[B27] Harris G. T. Skilling T. A. Rice M. E. (2001b). The construct of psychopathy. Crime Justice 28, 197–264

[B28] Harris G. T. Rice M. E. Quinsey V. L. (1993). Violent recidivism of mentally disordered offenders: the development of a statistical prediction instrument. Crim. Justice Behav. 20, 315–335 10.1177/0093854893020004001

[B29] Harris G. T. Rice M. E. Quinsey V. L. (1994). Psychopathy as a taxon: evidence that psychopaths are a discrete class. J. Consult. Clin. Psychol. 62, 387–397 10.1037/0022-006X.62.2.387 8201078

[B30] Harris G. T. Rice M. E. Quinsey V. L. Lalumière M. L. Boer D. Lang C. (2003). A multi-site comparison of actuarial risk instruments for sex offenders. Psychol. Assess. 15, 413–425 10.1037/1040-3590.15.3.413 14593842

[B31] Krupp D. B. DeBruine L. M. Barclay P. (2008). A cue of kinship promotes cooperation for the public good. Evol. Hum. Behav. 29, 49–55 10.1016/j.evolhumbehav.2007.08.002

[B32] Krupp D. B. DeBruine L. M. Jones B. C. (2011). “Cooperation and conflict in the light of kin recognition systems,” in The Oxford Handbook of Evolutionary Family Psychology, eds Salmon C. A. Shackelford T. K. (New York: Oxford University Press), 345–364

[B33] Krupp D. B. DeBruine L. M. Jones B. C. Lalumière M. L. (2012). Kin recognition: evidence that humans can perceive both positive and negative relatedness. J. Evol. Biol. 25, 1472–1478 10.1111/j.1420-9101.2012.02553.x 22694177

[B34] Lalumière M. L. Harris G. T. Quinsey V. L. Rice M. E. (2005). The Causes of Rape: Understanding Individual Differences in the Male Propensity for Sexual Aggression. Washington, DC: American Psychological Association

[B35] Lalumière M. L. Harris G. T. Rice M. E. (2001). Psychopathy and developmental instability. Evol. Hum. Behav. 22, 75–92 10.1016/S1090-5138(00)00064-7 11282307

[B36] Lalumière M. L. Mishra S. Harris G. T. (2008). “In cold blood: the evolution of psychopathy,” in Evolutionary Forensic Psychology, eds Duntley J. D. Shackleford T. K. (Oxford: Oxford University Press), 176–199

[B37] Lalumière M. L. Quinsey V. L. (1996). Sexual deviance, antisociality, mating effort, and the use of sexually coercive behaviors. Pers. Indiv. Differ. 21, 33–48 10.1016/0191-8869(96)00059-1

[B38] Lieberman D. Tooby J. Cosmides L. (2007). The architecture of human kin detection. Nature 445, 727–731 10.1038/nature05510 17301784 PMC3581061

[B39] Lynam D. R. Caspi A. Moffitt T. E. Loeber R. Stouthamer-Loeber M. (2007). Longitudinal evidence that psychopathy scores in early adolescence predict adult psychopathy. J. Abnorm. Psychol. 116, 155–165 10.1037/0021-843X.116.1.155 17324026 PMC3335348

[B40] McKelvey R. D. Zavoina W. (1975). A statistical model for the analysis of ordinal level dependent variables. J. Math. Sociol. 4, 103–120 10.1080/0022250X.1975.9989847

[B41] Mealey L. (1995). The sociobiology of sociopathy: an integrated evolutionary model. Behav. Brain Sci. 18, 523–599 10.1017/S0140525X00038589

[B42] Mokros A. Menner B. Eisenbarth H. Alpers G. W. Lange K. W. Osterheider M. (2008). Diminished cooperativeness of psychopaths in a prisoner’s dilemma game yields higher rewards. J. Abnorm. Psychol. 117, 406–413 10.1037/0021-843X.117.2.406 18489216

[B43] Nagelkerke N. J. D. (1991). A note on a general definition of the coefficient of determination. Biometrika 78, 691–692 10.1093/biomet/78.3.691

[B44] Nettle D. (2004). Evolutionary origins of depression: a review and reformulation. J. Affect. Disord. 81, 91–102 10.1016/j.jad.2003.08.009 15306134

[B45] Newman J. P. Curtin J. J. Bertsch J. D. Baskin-Sommers A. R. (2010). Attention moderates the fearlessness of psychopathic offenders. Biol. Psychiatry 67, 66–70 10.1016/j.biopsych.2009.07.035 19793581 PMC2795048

[B46] Penn D. J. Potts W. K. (1999). The evolution of mating preferences and major histocompatibility complex genes. Am. Nat. 153, 145–164 10.1086/303166 29578757

[B47] Pulkkinen L. Lyyra A. L. Kokko K. (2009). Life success of males on nonoffender, adolescence-limited, persistent, and adult-onset antisocial pathways: Follow-up from age 8 to 42. Aggress. Behav. 35, 117–135 10.1002/ab.20297 19184986

[B48] Pusey A. Wolf M. (1996). Inbreeding avoidance in animals. Trends Ecol. Evol. (Amst.) 11, 201–206 10.1016/0169-5347(96)10028-8 21237809

[B49] Quinsey V. L. Rice M. E. Harris G. T. (1995). Actuarial prediction of sexual recidivism. J. Interpers. Violence 10, 85–105 10.1177/088626095010001006

[B50] Rice M. E. Harris G. T. (1995). Psychopathy, schizophrenia, alcohol abuse and violent recidivism. Int. J. Law Psychiatry 18, 333–342 10.1016/0160-2527(95)00015-A 7591401

[B51] Rice M. E. Harris G. T. Cormier C. A. (1992). An evaluation of a maximum security therapeutic community for psychopaths and other mentally disordered offenders. Law Hum. Behav. 16, 399–412 10.1007/BF02352266

[B52] Richell R. A. Mitchell D. G. V. Newman C. Leonard A. Baron-Cohen S. Blair R. J. R. (2003). Theory of mind and psychopathy: can psychopathic individuals read the “language of the eyes”? Neuropsychologia 41, 523–526 10.1016/S0028-3932(02)00175-6 12559146

[B53] Scott-Phillips T. C. Dickins T. E. West S. A. (2011). Evolutionary theory and the ultimate-proximate distinction in the human behavioral sciences. Perspect. Psychol. Sci. 6, 38–47 10.1177/1745691610393528 26162114

[B54] Serin R. C. (1991). Psychopathy and violence in criminals. J. Interpers. Violence 6, 423–431 10.1177/088626091006004002

[B55] Tinbergen N. (1963). On aims and methods of ethology. Z. Tierpsychol. 20, 410–433 10.1111/j.1439-0310.1963.tb01161.x

[B56] Vachon D. D. Lynam D. R. Loeber R. Stouthamer-Loeber M. (2012). Generalizing the nomological network of psychopathy across populations differing on race and conviction status. J. Abnorm. Psychol. 121, 263–269 10.1037/a0024683 21842962 PMC3331676

[B57] Wakefield J. C. (1992). The concept of mental disorder — on the boundary between biological facts and social values. Am. Psychol. 47, 373–388 10.1037/0003-066X.47.3.373 1562108

[B58] Wheeler S. Book A. Costello K. (2009). Psychopathic traits and perceptions of victim vulnerability. Crim. Justice Behav. 36, 635–648 10.1177/0093854809333958

[B59] Williams G. C. (1966). Adaptation and Natural Selection. Princeton, NJ: Princeton University Press

[B60] Williamson S. Hare R. D. Wong S. (1987). Violence: criminal psychopaths and their victims. Can. J. Behav. Sci. 19, 454–462 10.1037/h0080003

[B61] Witt E. A. Hopwood C. J. Morey L. C. Markowitz J. C. McGlashan T. H. Grilo C. M. Sanislow C. A. Shea M. T. Skodol A. E. Gunderson J. G. Donnellan M. B. (2010). Psychometric characteristics and clinical correlates of NEO-PI-R fearless dominance and impulsive antisociality in the collaborative longitudinal personality disorders study. Psychol. Assess. 22, 559–568 10.1037/a0019617 20822268 PMC6838776

[B62] Wong S. (1988). Is Hare’s psychopathy checklist reliable without the interview? Psychol. Rep. 62, 931–934 10.2466/pr0.1988.62.3.931 3406310

